# British Medicinal Plants

**Published:** 1892-02

**Authors:** Alfred Heath

**Affiliations:** London, England


### BRITISH MEDICINAL PLANTS.

Alfred Heath, M. D., F. L. S., London, England.

### Order 37.—Umbelliferæ (Continued).

*Æthusa cynapium* (Fool's Parsley).—This plant is common in our gardens, and many persons have been poisoned by eating it in mistake for common parsley (*Petroselinum sativum*). It is easy to distinguish æthusa from petroselinum. The former has three long tail-like appendages or bracts depending from each little umbel. Æthusa is said to be good as a carminative to expel wind and relieve colic. It is diuretic, provoking urine, an emmenagogue, inducing the menses. There is a proving of this plant in Hering's *Guiding Symptoms.* It produces tearing, rending pains in the pit of stomach and abdomen, followed by nausea and vomiting, cramps in stomach, painful contractions, with cries and anguish, eructations, violent vomiting of frothy, curdy, milk-white substances. Pains in the kidneys with cutting in the bladder, with frequent urination or stoppage of urine. It suppresses the monthly period, producing lancinating pains.

*Pastinaca sativa* (Wild Parsnip).—Not much used in medicine, but is said to be diuretic and good against gravel and jaundice; it is also emmenagogue. The roots when *un*cultivated are said to be poisonous, but under cultivation they are sweet and wholesome, and highly nutritious. In some parts of Ireland the root of the parsnip is brewed instead of malt, and fermented ; the liquor obtained is said to be agreeable. The oil obtained from the seeds is said to cure some forms of intermittent fever. Parsnips are also grown for cattle-feeding ; animals grow very fat when fed on them.

*Heracleum sphondylium* (Cow-parsnip, All-heal).—Very common in our hedges. In Siberia this plant grows extremely high, and is there used as a remedy for dysentery. As long ago as the time of Galen this plant was esteemed as a remedy for cough with shortness of breath, epilepsy and jaundice. It has been used to remove the hard skin from fistula ; it has been found useful in removing lethargy, and stupors ; running scabs and shingles ; also in discharges from the ears. There is some proving in Allen's *Materia Medica ;* among other things it produces indolence, moist eruptions, etc.

*Daucus carota* (The Wild Carrot).—This plant is believed by most botanists to be the original stock from which the garden carrots have sprung, although Miller states that he in vain endeavored to improve the quality of the wild plant by cultivation.

The cultivated root of the carrot scraped and applied in the form of poultice is a well-known application for phagedænic ulcers, cancers, and putrid sores. The boiled root, although difficult to digest by weak stomachs, is very nutritious.

The wild carrot has been much esteemed as a remedy for flatulent colic, and to remove stitches in the side ; it is diuretic and emmenagogue ; it is said to break and expel stones from the bladder and kidneys; it is also said to help conception, and is useful in hysteria. I believe there is no proving of the plant, although it has been used somewhat in homœopathic practice.

*Chœrophyllum teniulum* (Rough Chervil).—This plant is said to make a very agreeable salad. Its medicinal virtues are many. It has been used with benefit in flatulent conditions, and in consumption of the lungs ; it is emmenagogue, and is said to promote expulsion of the placenta ; it increases appetite for meat ; the juice is healing if applied to ulcers ; it is said to protect against infection of various plagues ; it is perfectly harmless.

